# Phospholipid Fatty Acids as Physiological Indicators of *Paracoccus denitrificans* Encapsulated in Silica Sol-Gel Hydrogels

**DOI:** 10.3390/s150203426

**Published:** 2015-02-03

**Authors:** Josef Trögl, Ivana Jirková, Pavel Kuráň, Elmira Akhmetshina, Tat′jána Brovdyová, Alexander Sirotkin, Tatiana Kirilina

**Affiliations:** 1 Faculty of Environment, Jan Evangelista Purkyně University in Ústí nad Labem, KrálovaVýšina 3132/7, Ústí nad Labem 400 96, Czech Republic; E-Mails: IvanaJirkova@seznam.cz (I.J.); pavel.kuran@ujep.cz (P.K.); tatjana.brovdyova@ujep.cz (T.B.); 2 Faculty of Food Technology, Kazan National Research Technological University, Karl-Marx-Str. 8, Kazan 420 015, Russia; E-Mails: mira-axmetshina@mail.ru (E.A.); asirotkin@mail333.com (A.S.); tvkirilina@gmail.com (T.K.)

**Keywords:** phospholipid fatty acids, *cy*, *pre* indicator, encapsulation of bacteria, physiology of immobilized bacteria, sol-gel, whole-cell biosensing elements

## Abstract

The phospholipid fatty acid (PLFA) content was determined in samples of *Paracoccus denitrificans* encapsulated in silica hydrogel films prepared from prepolymerized tetramethoxysilane (TMOS). Immediately after encapsulation the total PLFA concentration was linearly proportional to the optical density (600 nm) of the input microbial suspension (R^2^ = 0.99). After 7 days this relationship remained linear, but with significantly decreased slope, indicating a higher extinction of bacteria in suspensions of input concentration 10^8^ cells/mL and higher. *trans*-Fatty acids, indicators of cytoplasmatic membrane disturbances, were below the detection limit. The *cy/pre* ratio (*i.e.*, ratio of cyclopropylated fatty acids (cy17:0 + cy19:0) to their metabolic precursors (16:1ω7 + 18:1ω7)), an indicator of the transition of the culture to a stationary growth-phase, decreased depending on co-immobilization of nutrients in the order phosphate buffer > mineral medium > Luria Broth rich medium. The ratio, too, was logarithmically proportional to cell concentration. These results confirm the applicability of total PLFA as an indicator for the determination of living biomass and *cy/pre* ratio for determination of nutrient limitation of microorganisms encapsulated in sol-gel matrices. This may be of interest for monitoring of sol-gel encapsulated bacteria proposed as optical recognition elements in biosensor construction, as well as other biotechnological applications.

## 1. Introduction

Optimized biocompatible sol-gel routes have recently enabled encapsulation of living cells into inorganic hydrogels forming biocomposite materials with interesting application properties [1–4]. Silica matrices, due to their transparency, are especially useful for preparation of recognition elements of optical biosensors, utilizing encapsulated bioluminescent or fluorescent bioreporters [5–8].

Despite optimization, different stresses are imposed on microorganisms both during the encapsulation procedure as well as in the final encapsulated state [1,9]. Determination of physiological state and viability of encapsulated microorganisms is therefore of significant importance; however the research in this field has been somewhat lagging behind the research of encapsulation procedures [1,10]. So far viability and stress of encapsulated microorganisms have been studied using many ways such as colony-forming units, various microscopy techniques, fluorescence, bioluminescence, metabolic and enzyme activities, or gene expression (see review [1] for details).

The physiological state of the microorganisms is among others manifested in the composition of its cytoplasmatic membrane via fast turnover of phospholipids and changes to the phospholipid fatty acid (PLFA) profile. PLFA profiling is routinely used for determinative purposes of pure microbial cultures and for rough characterization of soil microbial communities [11–14]. Recently we have demonstrated the usefulness of PLFA determination for characterization of viability of encapsulated microorganisms namely in polyvinyl alcohol (PVA) during biotechnological processes [15]. This preliminary study was however focused on total PLFA indicator only. In this study we aimed to test the applicability of this approach to microorganisms encapsulated in rigid silica matrix prepared by sol-gel method and also evaluate a few possible stress indicators (ratios of *trans/cis* PLFA and *cy/pre* PLFA) in response to nutrient insufficiency.

## 2. Experimental Section

### 2.1. Encapsulation

*Paracoccus denitrificans*, a strain used in previous studies [16–19], was obtained from LentiKat's a.s. (Prague, Czech Republic). Various types of silica films were prepared differing in the medium used for dilution of bacterial suspension prior to encapsulation and cell concentrations (Table 1). Cultivation was carried out on Bacterial Salt Medium (BSM [20]) with 1 g/L of glucose as a sole source of carbon and energy or in Luria Broth (Table 1) to an exponential growth-phase (OD_600_ = 0.2 to 0.5). Cells were harvested by centrifugation (4000 rpm, 10 min) and resuspended in an appropriate medium to the desired cell concentrations (Table 1).

Encapsulation in silica gels was carried out as described previously [8,21,22]. Briefly the tetramethoxysilane (TMOS) was prepolymerized overnight under acidic conditions (molar ratio TMOS:deionized water:HCl 1:5:10^−2^) at 4 °C. The formed sol (0.5 mL) was neutralized by NaOH (0.05 mol/L, 0.5 mL), mixed with cell suspension (2 mL) and poured on microscopy glass-slides. Slides were pretreated for better attachment of the films—wiped with toluene, acetone, and ethanol, immersed overnight in NaOH (1 mol/L), sonicated (20 min) in deionized water and dried (2 h, 120 °C). After gelification (∼1 min) the gel was immersed in phosphate buffer (50 mmol/L, pH 7). The gels were prepared in quadruplicates and stored under refrigerating conditions (4 °C) immersed in fresh sterile phosphate buffer (0.05 mol/L, pH 7).

### 2.2. Sampling and Analysis

Sampling was carried out on day 1 and day 7 after encapsulation. Four entire gels were withdrawn at once. Approximately half of the gel was used for moisture content determination and the rest was frozen (−40 °C) in Eppendorf tubes (1.5 mL) for further PLFA determination.

PLFA were determined as described previously [15]; a detailed procedure is provided in the Supplementary Material. Briefly, the total lipids from the sample of frozen gel were extracted using a single-phase mixture of chloroform, phosphate buffer, and methanol. Total lipids were fractionated to non-polar lipids, glycolipids and polar lipids. The polar lipid fraction was than subjected to mild alkaline methanolysis and the prepared fatty acids methyl esters (FAME) were determined by GC-MS using methyl nonadecanoate as internal standard. *trans/cis* PLFA indicator was calculated as the ratio (16:1ω7t + 18:1ω7t)/(16:1ω7 + 18:1ω7) [11]. *Cy/pre* indicator was calculated as the ratio (cy17:0 + cy19:0)/(16:1ω7 + 18:1ω7) [23].

## 3. Results and Discussion

### 3.1. Total PLFA Were Proportional to Input Biomass

Table 2 presents mutual correlations between input quantities of *Paracoccus denitrificans* in LB medium (measured as OD_600_ of the bacterial suspension used for encapsulation) and concentrations of total PLFA and several abundant FAMEs one day after encapsulation (gels A0 to A6). The highest correlation (r = 0.99) was obtained especially between OD_600_ and total PLFA which enabled linear regression (PLFA_tot_ = 1.0813OD_600_ + 0.1123, *n* = 12, R^2^ = 0.99). The intercept of this equation is significant (α = 0.05), *i.e.*, even gels with no input bacteria contained small but significant amounts of PLFAs. This phenomenon is likely related to the small amount of phospholipids in yeast extract, an important admixture of the Luria Broth medium used for dilution of the bacterial suspension. Such co-encapsulation of nutrients is important prerequisite for long-term survival of encapsulated microorganisms [1,24]. This also explains non-zero gel concentrations, uncorrelated to input biomass, of methyl linoleate (*cis*18:2ω6,9). This fatty acid is generally only produced by eukaryotes [11,25] and indeed it was not detected in a pure culture of the *P. denitrificans* strain as well as in further samples encapsulated without LB.

None of abundant single fatty acids was significantly correlated to input bacteria (α = 0.05) and the same was true even for their sum (Table 2). Such a result therefore disallows simple estimation of viable biomass concentrations based on a single FAME, leaving total PLFA concentration as a better indicator.

Repeated sampling of silica films seven days after encapsulation gave similar results; however the slope of the OD_600_ *versus* PLFA relationship decreased significantly (PLFA_tot_ = 0.0815×OD_600_ + 0.1113, *n* = 12, R^2^ = 0.82). The slope remained significant (α = 0.05), but the decrease indicates substantial extinction of encapsulated bacteria at input cell concentrations of order 10^8^ cells/g and higher; a phenomenon already observed in similar silica matrices [8,22,23].

### 3.2. Stress PLFA Indicators

Microorganisms need to maintain their cytoplasmatic membrane optimally permeable and fluid. As a result, membrane phospholipid fatty acids have fast turnover rate and reflect changes of the environment as well as in the cell physiology [11]. This led to the development of several PLFA-based stress indicators such as the ratio of *trans/cis* PLFA, branched/linear PLFA or *cy/pre* PLFA, widely used especially in soil ecology for studying of soil microbial communities [11,23].

The popular *trans/cis* PLFA ratio, a stress indicator of ongoing membrane transformations of dominant *cis* fatty acids to the corresponding *trans* isomers, could not be evaluated because the concentrations of indicator *trans* fatty acids 16:ω7t and 18:1ω7t were below the detection limit in all samples. This isomerization occurs in response to membrane perturbations and related stresses [11]. Such a negative result however indicates limited membrane interactions of the used encapsulation procedure and confirms its good biocompatibility.

**Figure 1.**
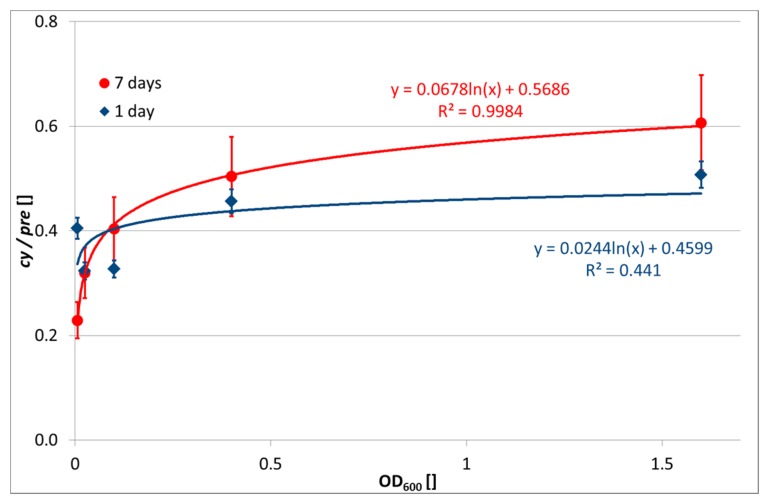
Relationship between biomass of *P. denitrificans* (expressed as OD_600_ of the input bacterial suspension) and *cy/pre* PLFA indicator at day 1 (blue diamonds) and day 7 (red circles) after encapsulation. Used gels A0 to A6.

In contrast, the evaluation of *cy/pre* ratio was successful. The abundance of cy17:0 and cy19:0 was high, as expected for the *Paracoccus* genus, since fatty acids with a cyclopropyl ring are typical for Gram-negative bacteria [12]. Increased transformation of monounsaturated fatty acids into cyclopropyl ones, known in Gram-negative bacteria, is observed upon transition of the culture into a stationary growth-phase as a response to nutrient insufficiencies and also stresses [11]. The relationship between OD_600_ of the input biomass and *cy/pre* ratio is depicted in Figure 1. While at day 1 after encapsulation there is a slightly increasing but very unclear relationship, at day 7 the *cy/pre* ratio shows a clear logarithmic dependence on bacterial concentration. This indicates a higher nutrition stress of higher bacterial concentrations.

**Figure 2.**
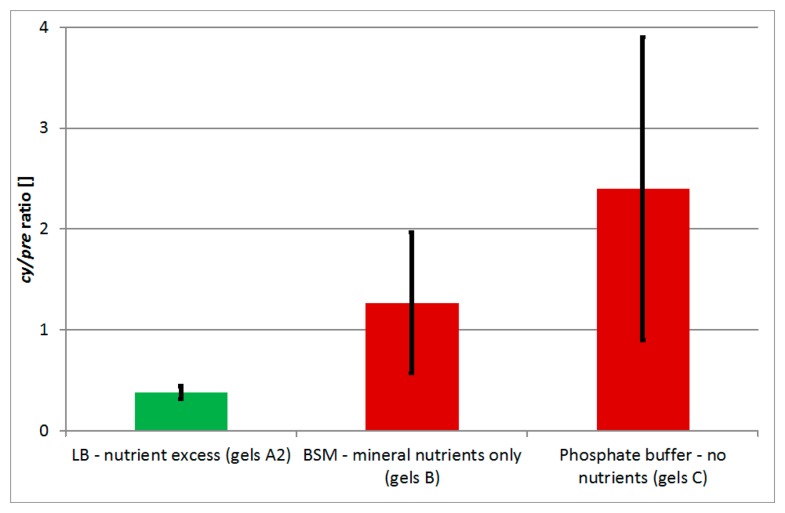
Values of *cy/pre* indicator as a function of three different media used for suspension of encapsulated *P. denitrificans* determined at day 1 after encgapsulation. Color indicates significant differences based on pair comparisons (t-test, α = 0.05). Error bars show 95%-confidence intervals.

In order to evaluate whether the increased value of *cy/pre* indicator indeed indicates nutrition stress, *P. denitrificans* was encapsulated with three variants of media used for bacterial suspension dilution and encapsulation, *i.e.*, phosphate buffer (imitating complete nutrient insufficiency, gels C), BSM (imitating lack of C-source but availability of mineral nutrients, gels B), and LB (imitating nutrients excess, gels A2). The values of *cy/pre* indicator increased in the order LB < BSM < phosphate buffer (Figure 2). Average values for bacteria encapsulated in BSM and phosphate buffer were significantly higher compared to LB (t-test, pair comparisons, α = 0.05), however they were mutually comparable. For *P. denitrificans* and likely for other bacteria, this result confirms the applicability of *cy/pre* indicator to detection of nutrient availability, especially to the C-source content.

### 3.3. Applications in Biosensor Construction

Sol-gel encapsulated microorganisms can find many biotechnological applications such as biocatalysis [26–28], production of hydrogen [29] or secondary metabolites [30,31]). Since such matrices, especially at low cell concentrations, are translucent, one of promising applications is the preparation of recognition elements for optical biosensors with encapsulated bioluminescent and fluorescent bioreporters [8,32,33]. Bioreporter response requires intact, metabolically active microbial cells and usually also supply of nutrients and oxygen. Failure to fulfill these conditions presents a risk of a false negative response. In such case PLFA analyses can provide useful information about the amount of living biomass as well as the nutrition stress (*cy/pre* ratio). The PLFA profile can be obtained in ∼2 days, far slower compared to biosensor responses (minutes to hours), which disqualifies the method for routine confirmation of negative results. Nevertheless PLFA data can be very useful in the design phase of the bioreporter especially during the optimization of encapsulation conditions and long-term verification of the function and stability.

### 3.4. Generalization

The obtained results demonstrate the applicability of PLFA profiles for assessment of the biomass and physiology of bacteria encapsulated in rigid inorganic silica matrices. Together with our previous study focused on bacteria encapsulated in polyvinyl alcohol [15], this indicates a wider applicability of this approach for monitoring of immobilized bacteria. Further verification with a wider spectrum of microorganisms, matrices and encapsulation procedures is however required.

## 4. Conclusions

This study demonstrated utilization of analyses of phospholipid fatty acids (PLFA) for estimation of the amount of living biomass of *Paracoccus denitrificans* encapsulated in a silica matrix prepared by sol-gel route from prepolymerized tetramethoxysilane. In addition, the PLFA profile enabled estimation of the nutrition stress of encapsulated bacteria via *cy/pre* indicator. Accounting previous study on polyvinyl alcohol-encapsulated bacteria the results indicate wider applicability of PLFA profiling for assessment of encapsulated microorganisms.

## Figures and Tables

**Table 1.** Setup of silica gel films.

**Label**	**Medium Used for**		**OD_600_** **of**	**Approx. Cell Conc.**
		
	**Cultivation**	**Dilution (Encapsulation)**	**Suspension**	**(CFU/mL)**
A0	LB	LB	0.00	0
A1	LB	LB	0.01	6 × 10^6^
A2	LB	LB	0.03	1 × 10^7^
A3	LB	LB	0.10	5 × 10^7^
A4	LB	LB	0.40	2 × 10^8^
A5	LB	LB	1.60	8 × 10^8^
B	BSM + glucose (1 g/L)	BSM	0.03	1 × 10^7^
C	BSM + glucose (1 g/L)	Phosphate buffer (0.05 mol/L, pH 7)	0.03	1 × 10^7^

Abbreviations: LB = Luria Broth, BSM = Bacterial Salt Medium [20], CFU = Colony forming units, OD_600_ = Optical density measured at λ = 600 nm

**Table 2.** Correlation between total PLFA, selected abundant FAME [mg.g^−1^ dry gel] and input biomass of encapsulated *P. denitrificans* (gels A0 to A6); *n* = 12, correlations significant at α = 0.05 are typed red bold-face.

	**Min**	**Max**	**Avg**	**Dev**	**1.**	**2.**	**3.**	**4.**	**5.**	**6.**	**7.**	**8.**
1. 16:0	0.006	0.019	0.012	0.005		**0.89**	−0.23	**0.90**	0.13	**0.92**	0.80	0.78
2. 18:1ω7	0.015	0.082	0.042	0.027	**0.89**		−0.61	**0.95**	0.29	**0.98**	0.64	0.58
3. 18:0	0.004	0.014	0.010	0.003	−0.23	−0.61		−0.37	−0.07	−0.46	0.17	0.25
4. cy19:0	0.005	0.028	0.013	0.008	**0.90**	**0.95**	−0.37		0.48	**0.99**	0.84	0.79
5. 18:2ω6,9	0.000	0.009	0.005	0.003	0.13	0.29	−0.07	0.48		0.41	0.56	0.52
6. Abundant	0.033	0.148	0.081	0.041	**0.92**	**0.98**	−0.46	**0.99**	0.41		0.78	0.73
7. PLFA_tot_	0.153	1.873	0.503	0.679	0.80	0.64	0.17	0.84	0.56	0.78		**0.99**
8. OD_600_	0.000	1.600	0.360	0.630	0.78	0.58	0.25	0.79	0.52	0.73	**0.99**	

Min—Minimal value, Max—maximal value, Avg.—average value, Dev.—Standard deviation. 1. 16:0-methyl hexadecanoate (palmitate); 2.18:1ω7-methyl *cis-*11-octadecenoate (vaccenate); 3. 18:0-methyl octadecanoate (stearate); 4. cy19:0-methyl *cis-*9,10-methylenoctadecanoate; 5. 18:2ω6,9-methyl *cis-*9,12 octadecedieoate (linoleate); 6. Abundant-Sum of concentrations of previous abundant FAME (16:0 + 18:1ω7 + 18:0 + cy19:0); 7. Total PLFA—sum of PLFA concentrations; 8. OD_600_—Optical density measured at α = 600 nm.
